# Metformin attenuates trauma‐induced heterotopic ossification via inhibition of Bone Morphogenetic Protein signalling

**DOI:** 10.1111/jcmm.16076

**Published:** 2020-11-10

**Authors:** Hui Lin, Fuli Shi, Shanshan Jiang, Yuanyuan Wang, Junrong Zou, Ying Ying, Deqiang Huang, Lingyu Luo, Xiaohua Yan, Zhijun Luo

**Affiliations:** ^1^ Jiangxi Province Key Laboratory of Tumor Pathogenesis and Molecular Pathology and Department of Pathophysiology School of Basic Medical Sciences Queen Mary School, Nanchang University Nanchang China; ^2^ Institute of Hematological Research Shaanxi Provincial People's Hospital Xi'an China; ^3^ Clinical Systems Biology Laboratory The First Affiliated Hospital Zhengzhou University Zhengzhou China; ^4^ Research Institute of Digestive Diseases The First Affiliated Hospital Nanchang University Nanchang China; ^5^ Institute of Basic Biomedical Sciences and Department of Biochemistry and Molecular Biology School of Basic Medical Sciences Nanchang University Nanchang China

**Keywords:** AMP‐activated protein kinase, BMP signalling pathway, metformin, trauma‐induced heterotopic ossification

## Abstract

AMP‐activated protein kinase (AMPK) is an intracellular sensor of energy homoeostasis that is activated under energy stress and suppressed in energy surplus. AMPK activation leads to inhibition of anabolic processes that consume ATP. Osteogenic differentiation is a process that highly demands ATP during which AMPK is inhibited. The bone morphogenetic proteins (BMPs) signalling pathway plays an essential role in osteogenic differentiation. The present study examines the inhibitory effect of metformin on BMP signalling, osteogenic differentiation and trauma‐induced heterotopic ossification. Our results showed that metformin inhibited Smad1/5 phosphorylation induced by BMP6 in osteoblast MC3T3‐E1 cells, concurrent with up‐regulation of Smad6, and this effect was attenuated by knockdown of Smad6. Furthermore, we found that metformin suppressed ALP activity and mineralization of the cells, an event that was attenuated by the dominant negative mutant of AMPK and mimicked by its constitutively active mutant. Finally, administration of metformin prevented the trauma‐induced heterotopic ossification in mice. In conjuncture, AMPK activity and Smad6 and Smurf1 expression were enhanced by metformin treatment in the muscle of injured area, concurrently with the reduction of ALK2. Collectively, our study suggests that metformin prevents heterotopic ossification via activation of AMPK and subsequent up‐regulation of Smad6. Therefore, metformin could be a potential therapeutic drug for heterotopic ossification induced by traumatic injury.

## 1 INTRODUCTION

Adenine monophosphate‐activated protein kinase (AMPK) is a conserved serine/threonine protein kinase, consisting of three subunits: α (α1, α2), b (β1, β2) and γ (γ1, γ2, γ3).^1^, ^2^ AMPK acts as a fuel‐sensing enzyme that is suppressed in energy surplus and activated in response to energy stress such as hypoxia, glucose deprivation, ischaemia and oxidative stress. Under the stress condition, an increase in intracellular AMP or the ratio of AMP to ATP allows AMP directly binds to and allosterically activates AMPK. Recently, an AMP‐independent activation of AMPK has been delineated which involves lysosomes under glucose deprivation.^3^, ^4^ The activation of AMPK attenuates anabolism and stimulates catabolism, resulting in preservation of energy for acute cell survival programme and generation of more ATP to cope with stress. As AMPK activation enhances glucose uptake and insulin sensitivity, it is a well‐received drug target for metabolic syndrome, type 2 diabetes and cancer.^5^

Metformin, a widely used oral drug for type 2 diabetes mellitus, has been reported to have multiple functions. The action of metformin includes inhibition of hepatic gluconeogenesis and enhancement of insulin sensitivity by stimulating glucose uptake in muscle and adipose.^6^ It is known that many effects of metformin are mediated by AMPK. Furthermore, pre‐clinical and clinical studies have shown that metformin is a promising drug as an adjuvant or neoadjuvant agent for cancer therapy.^7^, ^8^, ^9^ Over the last decade, metformin has been documented to regulate osteogenic differentiation through AMPK‐dependent and independent manner.^10^, ^11^ AMPK is inhibited through the increased glucose uptake during the differentiation, an event that is suppressed by metformin.^12^, ^13^

Heterotopic ossification (HO) refers to aberrant formation of extra‐skeletal bone in muscle, tendons and ligaments, which often occur after musculoskeletal trauma, severe burns and hip arthroplasty.^14^ HO causes chronic pain and loss of joint mobility and severely impairs the quality of life.^15^ At present, there is no effective management for HO, except that non‐steroid anti‐inflammatory drugs (NSAID) and local irradiation are used for prophylaxis.^16^ The only option is to surgically resect the symptomatically established heterotopic bone. However, the risk of ectopic bone recurrence is even greater after surgical excision.^17^, ^18^ Consequently, there is unmet need for prophylaxis and treatment of HO. A critical step is to identify key targets implicated in HO and then develop intervention approaches.

The bone morphogenetic proteins (BMPs) signalling pathway plays an essential role in osteogenic differentiation and osteo‐induction.^17^, ^19^ BMPs bind to the type I and type II receptor complex, triggering the type II receptor to phosphorylate and activate the type I receptor, which then elicits Smad and non‐Smad signalling cascades via phosphorylation events that regulate transcription of target genes. Smad1/5/8 forms a complex with Smad4 upon phosphorylation by the type I receptor, and the complex then translocates into the nucleus and activates the transcription programme.^20^ Smad6 is a negative regulator specific to the BMP signalling. It disrupts the interaction between activated type I receptor with Smad1/5/8 and/or induces their degradation via ubiquitination pathway when it recruits Smurf1.^12^, ^21^, ^22^

The BMP signalling pathway is also involved in many human diseases, particularly in HO formation.^23^ For example, fibrodysplasia ossificans progressiva (FOP), a rare genetic disorder characteristic of progressive HO formation, arises from mutations of the BMP type I receptor ALK2, 95% of which are R206 to H mutation resulting in a constitutive activation of the receptor and Smad1/5‐mediated signalling events essential for osteogenesis.^24^ Furthermore, the BMP signalling pathway is often aberrantly activated by inflammation secondary to traumatic injury and contributes to pathogenic HO.^19^, ^25^ BMP2 has also been shown to trigger HO in animal models.^25^, ^26^, ^27^ An innovative strategy would be to identify drugs already used in clinic for other diseases, which can block the BMP signalling pathway and prevent HO formation. Hence, they could immediately enter clinical translation such that tedious steps to characterize their adverse effects could be curtailed.

Recently, we have shown that AMPK inhibits the activity of ALK2 R206H mutant identified from FOP, concurrent with suppression of osteogenic differentiation of induced pluripotent stem cells derived from an FOP patient fibroblast.^28^ The present study further tests if metformin can generate similar results in different osteoblast cells and traumatic HO animal model.

## 2 MATERIALS AND METHODS

### 2.1 Reagents

BCIP/NBT Liquid Substrate System, metformin and ibuprofen were purchased from Sigma‐Aldrich (St. Louis, USA). Alizarin red S staining kit and Mesenchymal Stem Cell Osteogenic Differentiation Medium (MSCODM)were purchased from Cyagen Biosciences (Guangzhou, China). Antibodies and recombinant mouse BMP6 were listed in Table 1.

**Table 1:** List of antibodies and protein used in this study

Antibody/protein	Catalog number	Vendor	City and Country
AMPKα Antibody	2532L	Cell Signaling Technology	Danvers, USA
Phospho‐AMPKα(Thr172) (D4D6D) Rabbit mAb	#50081	Cell Signaling Technology	Danvers, USA
Smad1 (D59D7) XP® Rabbit mAb	6944S	Cell Signaling Technology	Danvers, USA
Phospho‐Smad1/5 (Ser463/465) (41D10) Rabbit mAb	9516S	Cell Signaling Technology	Danvers, USA
ACTR‐I Antibody (C‐5)	sc‐374523	Santa Cruz Biotechnology	Dallas, USA
Smurf1 Antibody (45‐K)	sc‐100616	Santa Cruz Biotechnology	Dallas, USA
Anti‐beta Actin antibody	ab8227	Abcam	Cambridge, USA
Anti‐SMAD6 antibody	ab214009	Abcam	Cambridge, USA
Recombinant mouse BMP6 protein	6325‐BM/CF	R&D System	Minneapolis, USA

### 2.2 Cell culture and transfection

The MC3T3‐E1 cell line (CRL 2593 subclone 4) was purchased from American Type Culture Collection (Manassas, USA) and cultured in Eagle's minimum essential medium alpha (α‐MEM) supplemented with 10% Fetal Bovine Serum (FBS) and 1% penicillin and streptomycin at 37°C in a humidified atmosphere supplied with 5% CO2. siRNAs were transfected into the MC3T3‐E1 cells with Lipofectamine 2000 according to instructions provided by the manufacturer. Negative control siRNAs (AM4611) and Smad6 siRNAs (4 390 771) were purchased from Life Technologies (Carlsbad, USA).

### 2.3 Adenovirus preparation and cell infected

Adenovirus expressing the constitutively active or dominant negative mutant of AMPKα1 was prepared as described previously.^29^ Adenovirus expressing GFP was prepared as a control. The multiplicity of infection was determined after virus preparation according to standard method. The recombinant AMPK was tagged by a flag epitope. MC3T3 E1 cells were infected with adenovirus encoding the active mutant of AMPK (Ad‐AMPK‐CA), dominant negative mutant of AMPKα1 or GFP (Ad‐GFP) for 48 hours, and then, osteogenic differentiation was induced.

### 2.4 Osteogenic differentiation assays

Osteogenic differentiation was induced in the MSCODM, which was changed every 3 days. After 7 days, the ALP staining was performed using the BCIP/NBT Liquid Substrate kit according to the protocol provided by manufacturer. In brief, the cells were washed with PBS and then incubated with ALP staining solution at room temperature for 2 hours. For Alizarin red s staining, the cells were fixed after 21‐day differentiation, stained with Alizarin red s staining solution in dark at room temperature for 30 minutes and washed with distilled water. Finally, the stained cells were photographed under a light microscope after colour development.

For quantification, ALP deposit was dissolved in 10% (w/v) cetylpyridinium chloride (Sigma‐Aldrich, St. Louis, USA) in 10 mM sodium phosphate (pH 7.0) and quantified at 540 nm absorbance. Alizarin deposit was dissolved in solution containing 0.5 N HCL and 5% SDS and read at 405 nm absorbance.

### 2.5 Trauma/burn induced heterotopic ossification mouse model

C57BL/6 female mice were purchased through the Center of Laboratory Animal Science of Nanchang University. Animal protocol was reviewed and approved by the Animal Care Committee of Nanchang University (Animal protocol: NCDXSYDWFL‐2015097). This study was performed in accordance with National Institutes of Health Guide for Animal Care. Trauma/burn induced HO model was generated by performing Achilles tenotomy plus burn injury, as previously described.^30^, ^31^ Briefly, 8 weeks old female C57BL/6 mice were subjected to a right hindlimb Achilles tenotomy under general anaesthesia with intraperitoneal injection of chloral hydrate (300mg/kg) and subsequent burn injury on the dorsum. A 60°C metal block was applied to the dorsum for 20 seconds. All mice were monitored and kept under standard conditions. Next day after surgery, mice were treated with metformin (100mg/kg, *i.p*.) in PBS or PBS as vehicle every 3 days for 8 weeks (vehicle = 6, metformin = 5). The health and well‐being of mice were monitored during the study. The CO_2_ was used to kill mice at the end of experiment.

### 2.6 Micro‐computerized tomography analysis

Eight weeks after Achilles tenotomy, the injured hindlimbs were collected after sacrificing animals. Trauma induced HO was assessed with MicroCT scans (μCT 100, Scanco Medical AG, Switzerland) through customer service provided by Hangzhou Yuebo Biological Technology Co., Ltd (Hangzhou, China). The parameters were as follows: 70kv (voltage), 200uA (current), 300ms (exposure time), and 14.5 μm (per pixel). Two‐dimensional images were acquired, and the 3D images automatically reconstructed with Evaluation V6.5‐3 software (SCANCO Medical AG). After reconstruction, the heterotopic ossification volume was quantified according to the calibrated imaging protocol as previously described using Evaluation V6.5‐3 software(SCANCO Medical AG).^32^ HO volumes were calculated at threshold 200 for all samples. A phantom was used for normalization. The region of interest (ROI) was defined for HO formation in soft tissues and segmented based on anatomical landmarks, Hounsfield units, and observer identification.

### 2.7 Histological analysis

Eight weeks post‐injury, the hindlimbs were collected after euthanization and fixed in 4% phosphate buffered paraformaldehyde for 48 hours. Decalcification of samples was achieved in 19% EDTA solution at 4°C for 4 weeks and then paraffin embedded. A series of 4 µm sections were processed for haematoxylin and eosin (H&E) staining. The H&E staining was performed using the H&E staining kit (Solarbio, Beijing, China) according to the manufacturer's instructions.

### 2.8 Protein extraction and Western Blot

Total proteins isolated from injured tissue or cells were prepared in RIPA lysis buffer plus protease inhibitors. The extracts were centrifuged at 12 000×g, 4°C for 15 minutes. Western blot was conducted using the standard protocol as described previously.^28^ Equal amounts of proteins (20 μg) were resolved onto SDS‐PAGE and transferred to PVDF membranes (Millipore Sigma). The membranes were incubated with primary antibodies at 4°C overnight and then HRP‐conjugated secondary antibodies (Thermo Fisher Scientific) at room temperature for 1 hour. The blot signals were detected with ECL reagent.

### 2.9 Statistical analysis

Data were analysed using GraphPad Prism software. All quantitative data were calculated as the mean ± standard deviation (SD). Difference between groups was assessed with Student's t test, and *P* < .05 was set for significance.

## 3 RESULTS

### 3.1 Metformin reduces BMP signalling

**Figure 1:**
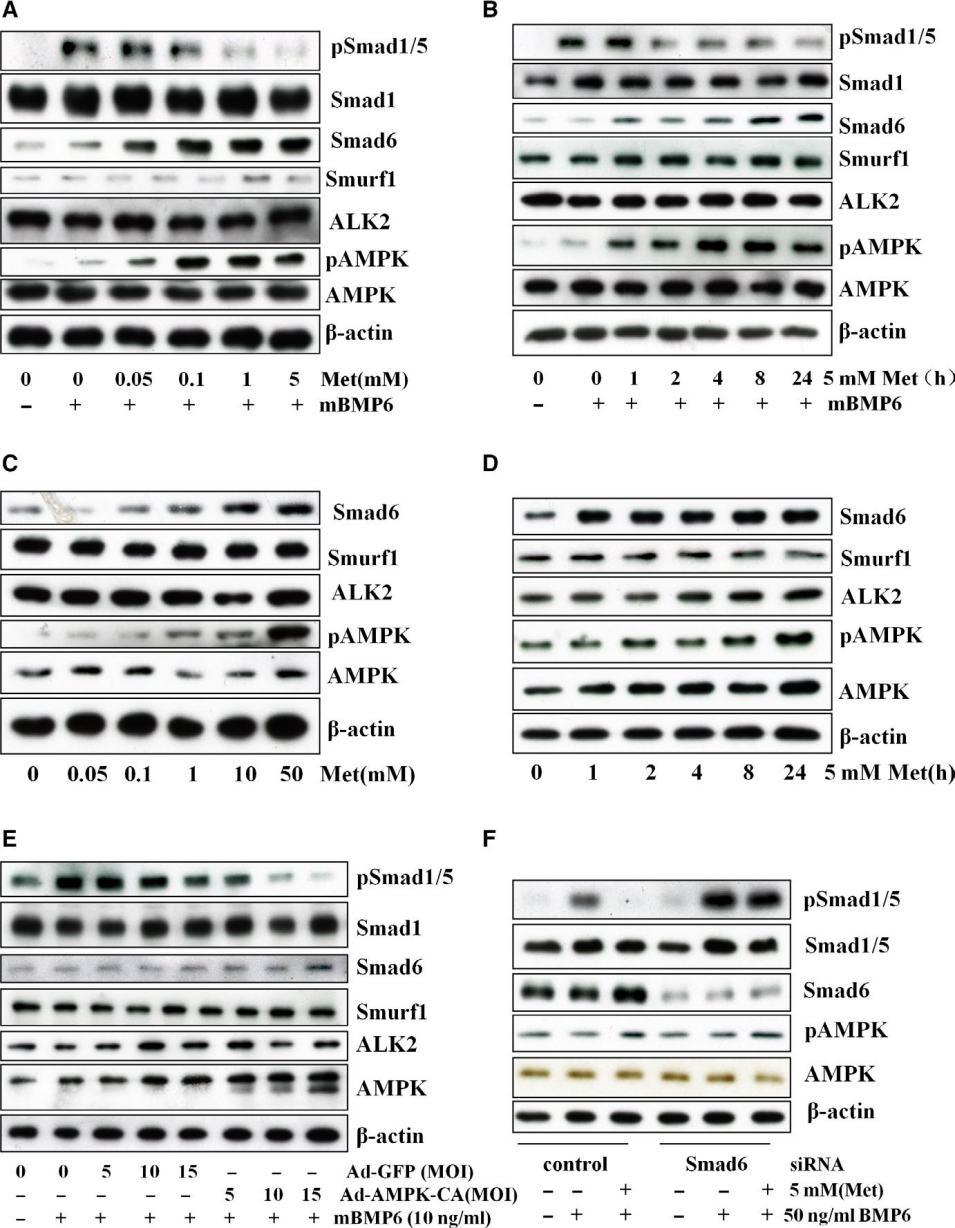
Metformin reduces BMP signalling pathway in MC3T3‐E1 cells. MC3T3‐E1 osteoblast cells were treated with metformin(Met) at different doses for 24 hours (A), or at 5 mM for different time (B), followed by BMP6 (50ng/ml) for 30 min. C&amp;D. MC3T3‐E1 cells were treated the same as (A) and (B), but without BMP6. E. MC3T3 E1 cells were infected with adenovirus encoding the active mutant of AMPK (Ad‐AMPK‐CA) or GFP (Ad‐GFP) at different MOI (5, 10 and 15), and immunoblot was performed with antibodies as indicated after 48 hours. F.MC3T3‐E1 cells were transfected with Smad6 siRNA and control siRNA, and treated with metformin (5 mM) for 24 hours, followed by mouse BMP6 (50 ng/mL) for 30 min. Immunoblot was performed. Each experiment was repeated three times, and the representative blot was shown

To explore the effect of metformin on BMP signalling pathway, MC3T3‐E1 cells were treated with metformin at different doses or for different hours, and then followed with BMP6 for 30 minutes. The results revealed that BMP‐induced Smad1/5 phosphorylation was markedly offset by metformin (Figure 1A&B), which was associated with the induction of AMPK phosphorylation at Thr172, an indicator of the activation. Further, our data showed that metformin up‐regulated Smad6, but without changes in Smurf1 and ALK2. To exclude whether the increased expression of Smad6 was not induced by BMP6, we repeated the same experiments only with metformin. The results clearly revealed that Smad6 was induced by metformin (Figure 1C&D). To ascertain whether AMPK mediates the effect of metformin, we used the adenovirus expressing a constitutively active mutant of AMPK or GFP as a control. Our study revealed that the infection with the active form of AMPK progressively reduced phosphorylation of Smad1/5 induced by BMP6, concomitant with elevation of Smad6 (Figure 1E). Next, we transfected Smad6 siRNA into MC3T3‐E1 cells and found that knockdown of Smad6 blunted the inhibitory effect of metformin (Figure 1F). Altogether, our results demonstrated that metformin via activation of AMPK up‐regulated Smad6 and thus suppressed BMP‐Alk2‐Smad1/5 signalling event.

### 3.2 Metformin suppresses osteoblast differentiation

**Figure 2:**
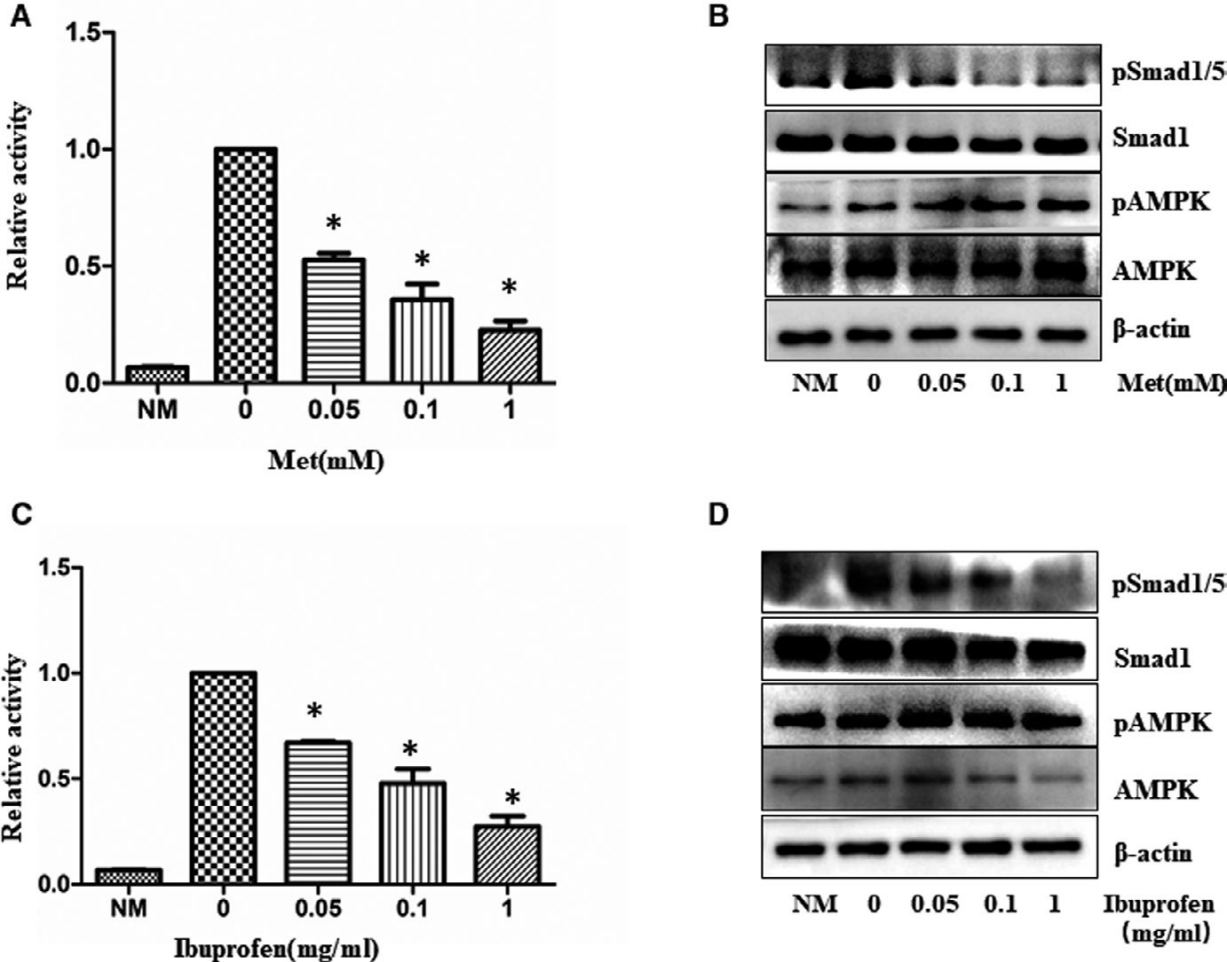
AMPK activators inhibit alkaline phosphatase activity during osteogenic differentiation. MC3T3‐E1 cells were incubated with osteogenic differentiation medium in the presence of different doses of metformin (Met) and Ibuprofen. After one week, the Alkaline phosphatase activity (metformin (Met) (A) and Ibuprofen(C)) was measured. The graph represents averages of three independent experiments (mean ± SD, N = 3). *Significant difference from metformin (A) and Ibuprofen(C) versus control was assessed using Student's t test (*P < .05). Western blot (metformin (B) and Ibuprofen(D)) was also performed to study activation of AMPK and Smad1/5. NM: no differentiation medium, α‐MEM complete medium

We found that AMPK activity was inhibited during the course of osteogenic differentiation of MC3T3‐E1 cells (Supplementary Figure S1). This finding together with those shown above suggests that forced AMPK activation could suppress osteogenic differentiation by either pharmacological agents or its active mutant. To test this hypothesis, we cultured the MC3T3‐E1 cells in the differentiation medium (MSCODM) in absence or presence of different doses of AMPK activators, metformin and ibuprofen. Western blots and assays on alkaline phosphatase activity (ALP) were performed after 7 days. The data revealed that AMPK activators progressively decreased ALP activity in a dose‐dependent manner in parallel to activation of AMPK and suppression of Smad1/5 phosphorylation (Figure 2). Interestingly, p‐Smad1/5 signal was remarkably higher than that without differentiation induction, suggesting p‐Smad1/5 is involved in the regulation of ALP activity.

**Figure 3:**
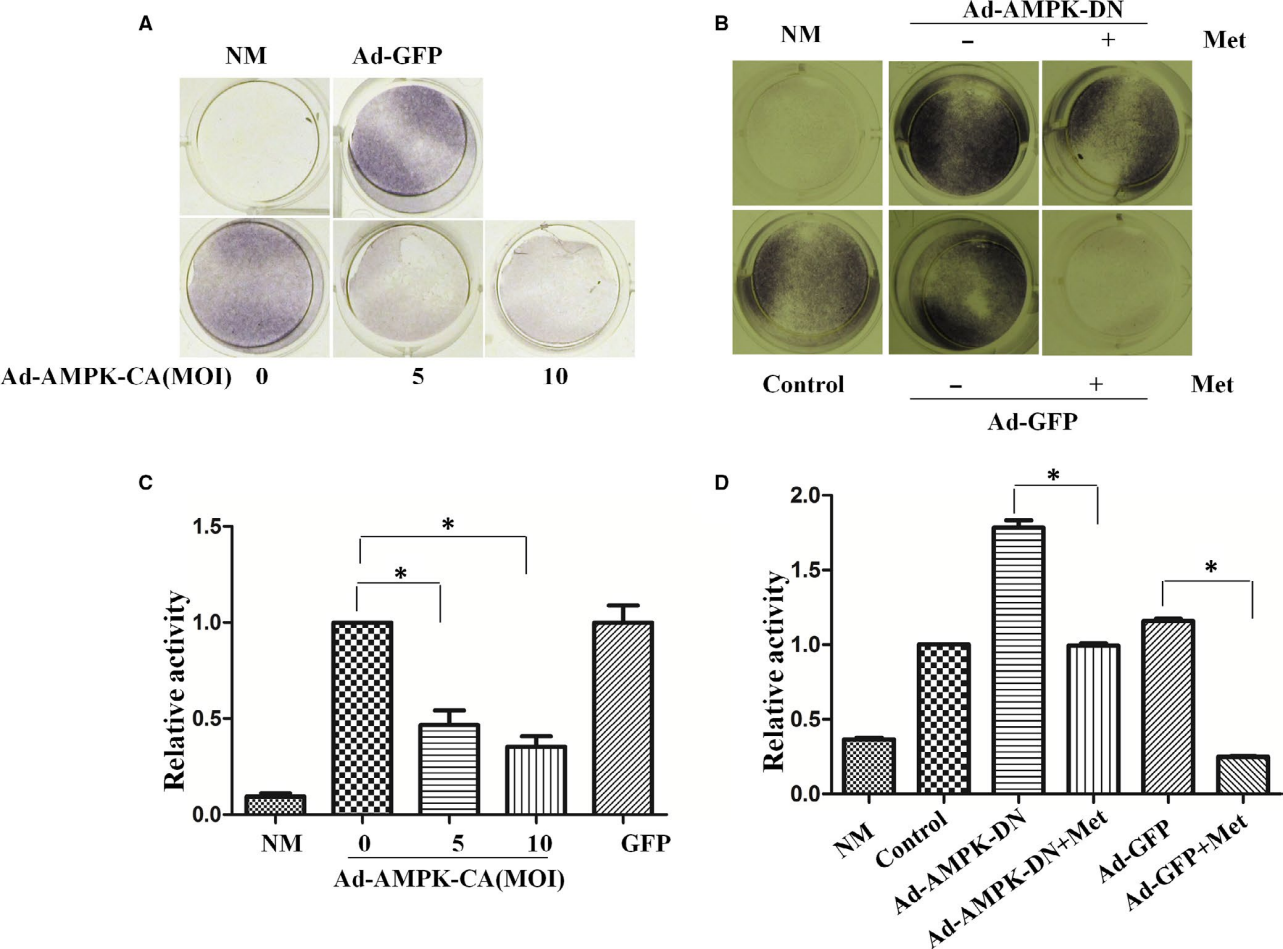
AMPK mediates the inhibitory effect of metformin on alkaline phosphatase activity. A&amp;B. MC3T3‐E1 cells were infected with varying multiplicity of infection (MOI) of adenovirus expressing a constitutively active mutant of AMPK α 1 (Ad‐AMPK‐CA) (A), or the dominant negative mutant (AMPK‐DN) (B) for 48 hours and then were incubated with osteogenesis differentiation media in the presence or absence of 1mM metformin (Met). GFP adenovirus was used as a control. Alkaline phosphatase activity was measured one week after the induction. C&amp;D. The graph represents averages of 3 independent experiments from A and B, respectively (mean ± SD, N = 3). Statistical analysis was performed using Student's t test, and p values between groups were indicated (*P < .05). NM, no differentiation media, α‐MEM complete medium

To clarify the mechanisms by which the metformin inhibits ALP, we pre‐infected the cells with the constitutively active (Figure 3A) or dominant negative form of AMPK (Figure 3B), then replaced the culture medium with the differentiation medium and cultured the cells for additional 7 days. Our study revealed that ALP activity was suppressed by the active AMPK mutant, and no effects were found in GFP adenovirus infection. In contrast, the inhibitory effect of metformin on ALP activity was partially blocked by the dominant negative mutant AMPK. The quantification of ALP staining gave consistent results (Figure 3C&D). The partial blocking effect of the dominant negative AMPK suggests two possibilities: first, its amount was not sufficient to compete with endogenous wild‐type counterpart, and second, metformin takes effect through both AMPK‐dependent and independent mechanisms.

**Figure 4:**
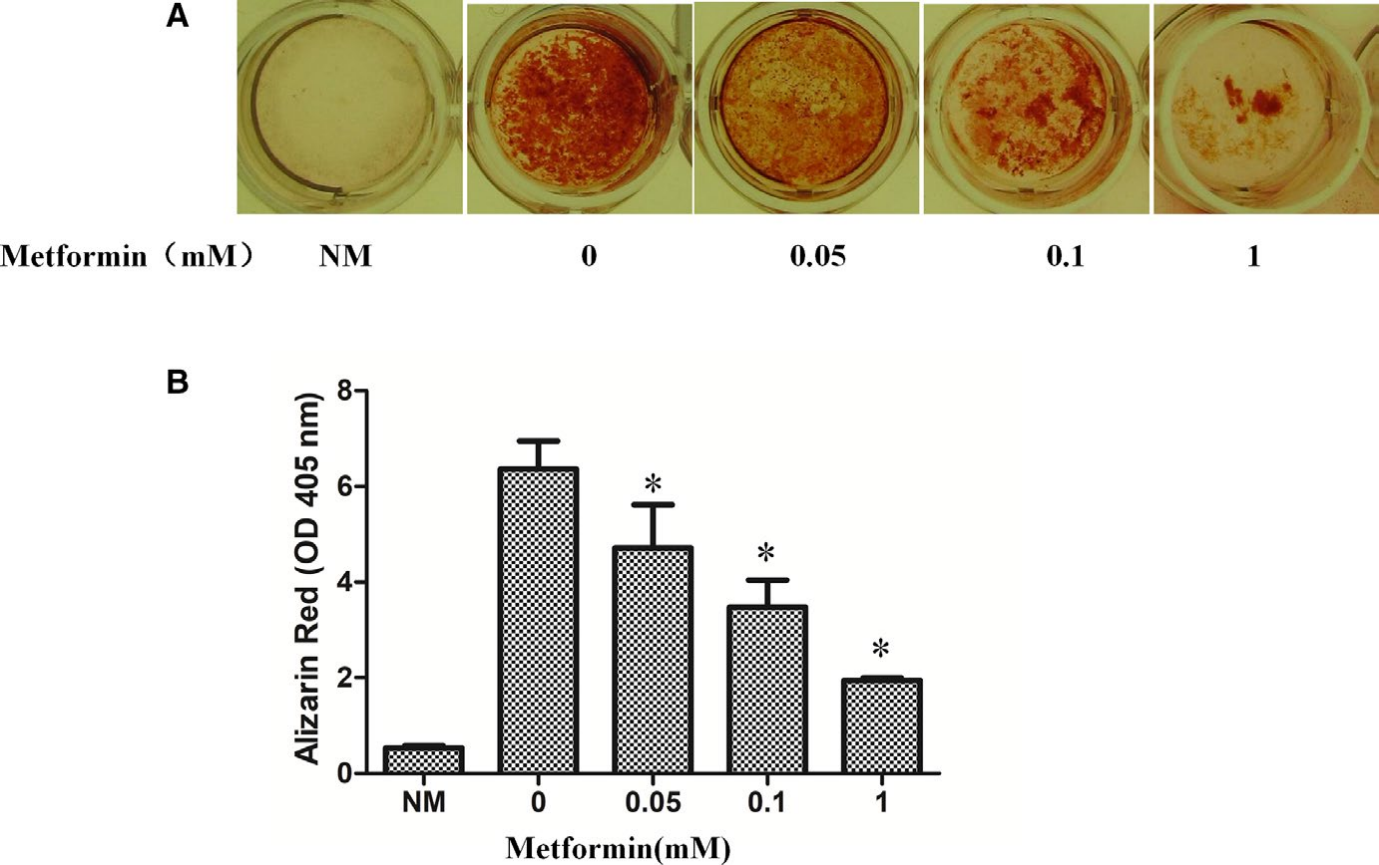
The effect of metformin on mineralization. MC3T3‐E1 cells were incubated with osteogenesis differentiation media in the presence or absence of varying doses of metformin for 21 days and stained with alizarin red. A. Representative images of differentiated cells. B. Graph represents alizarin deposit quantitation from 3 independent experiments (mean ± SD, N = 3). *Significant difference from metformin versus control was assessed by Student's t test (*P < .05). NM: no differentiation media, α‐MEM complete medium

Finally, to assess whether AMPK affects mineralization, the late stage of osteoblast differentiation. As expected, the results showed that metformin significantly suppressed mineralization (Figure 4). Taken together, the study suggests that osteogenic differentiation of MC3T3‐E1 cells is suppressed by AMPK activation. At present, we are not certain if AMPK interferes with early or late stage of differentiation, as the manoeuvre with exogenous agents was conducted at the beginning of differentiation.

### 3.3 Metformin inhibits trauma‐induced heterotopic ossification

**Figure 5:**
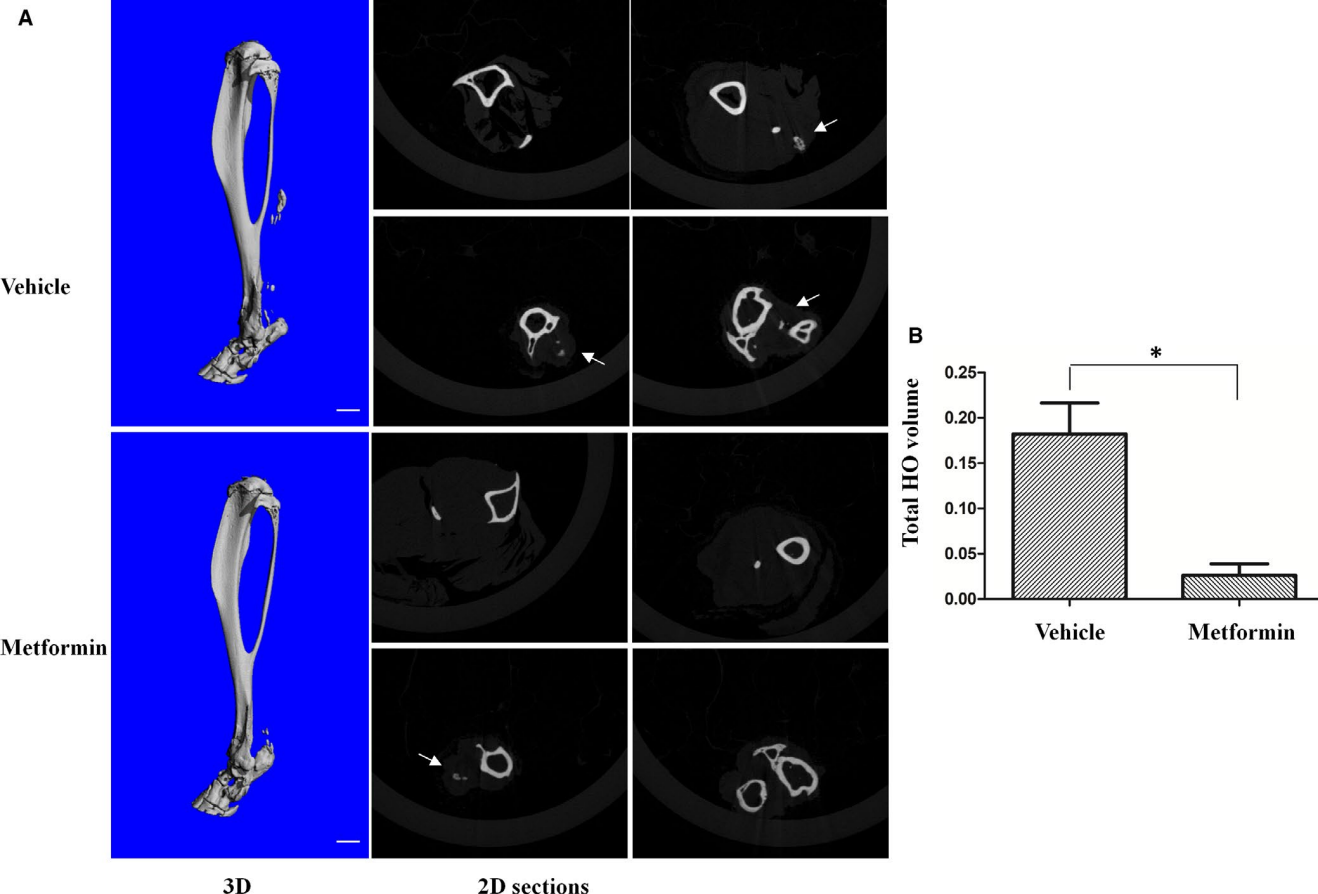
Metformin prevents trauma‐induced HO in mice. A. Mice received Achilles tenotomy on the right hindlimb and burn injury on the dorsum. The next day, they were injected*i.p*.with vehicle (PBS) or metformin every 3 days for 8 weeks (vehicle, n = 6, metformin, n = 5). The injured limbs were examined by microCT at 8 weeks post‐injury. Representative 3D microCT reconstructions and serial 2D cross‐sections are shown in Figure A. Scale bar = 1mM, white arrows indicate ectopic bone. (B). The volume of HO was quantified from each group. * Statistical analysis was performed using Student's t test, and p values between metformin‐treated and vehicle‐treated group were indicated (*P < .05; vehicle, n = 6; metformin, n = 5)

**Figure 6:**
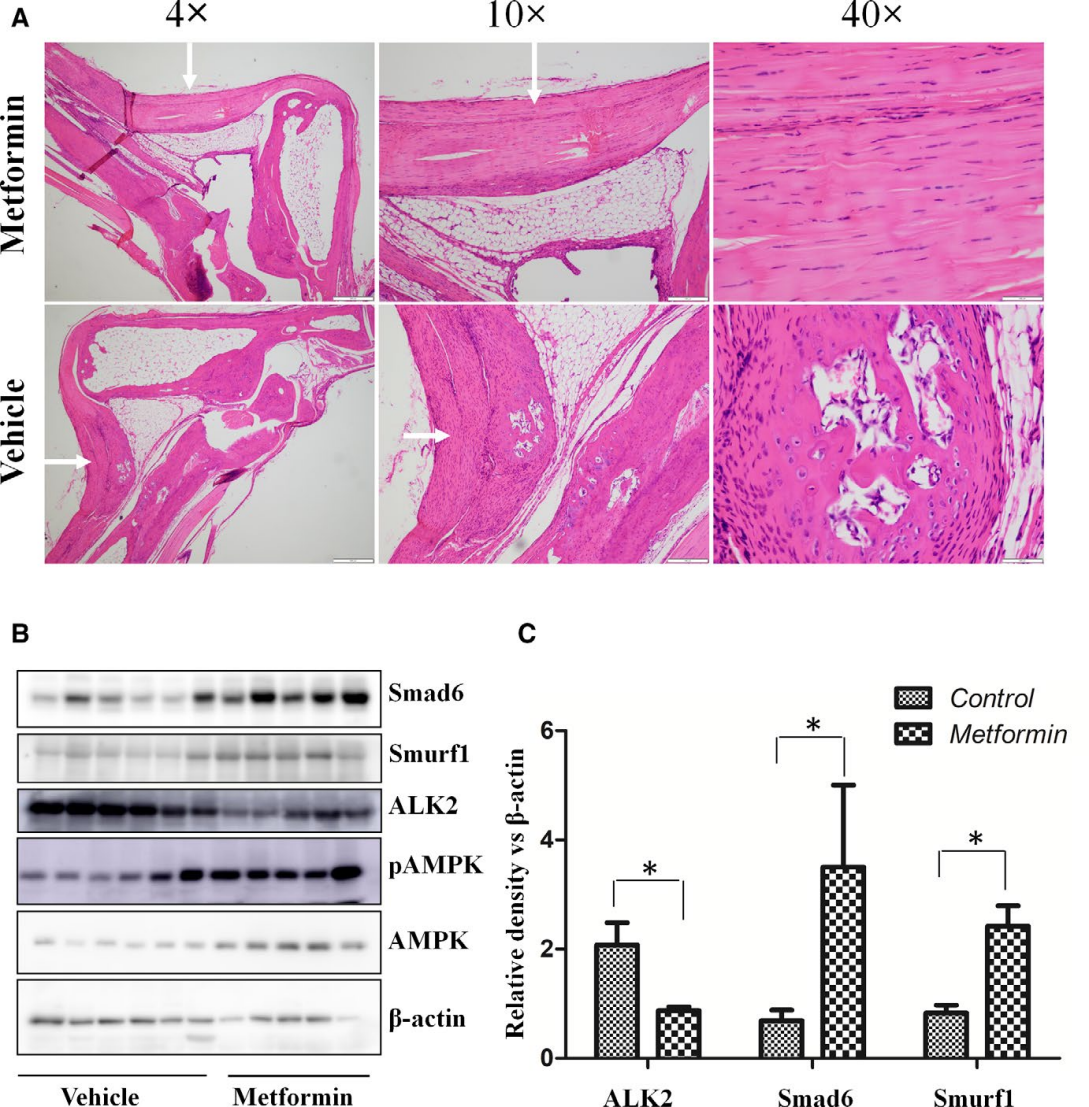
Effect of metformin on histological and molecular changes in the trauma‐induced HO. A. Representative H&amp;E staining of HO at 8 wk after trauma. The injured tissues collected from vehicle‐treated control and metformin‐treated mice were sectioned and examined by H&amp;E (vehicle, n = 6; metformin, n = 5). Areas of interest in sections were examined at different magnification, and the scale bar is indicated. B. Effect of metformin on the expression of Smad6, ALK2 and Smurf1 in injured tissue. Western blot was performed with antibodies, as indicated. C. Graphs represent scan densitometric ratio of bands from B. Statistical analysis was performed using Student's t test, and p values between metformin‐treated and vehicle‐treated group were indicated, *P < .05, (vehicle, n = 6; metformin, n = 5)

To determine whether metformin prevents HO induced by trauma, mice received Achilles tenotomy and burn in dorsum. One day after trauma, mice were treated with metformin (100 mg/kg, *i.p*.) every 3 days for 8 weeks or PBS as vehicle control. There were no systemic side effects after treatment (data not shown). Injured Achilles were removed and processed for microCT scan. As shown in Figure 5A, obvious formation of ectopic bone was observed in the area of the tenotomy of the mice receiving vehicle treatment, but it was significantly reduced in metformin‐treated mice. The volume of total HO was calculated with software and revealed a marked reduction after metformin treatment as compared to the vehicle control (Figure 5B). In consistency, HE staining further demonstrated ectopic bone, bone matrix, bone marrow and abundant cartilage in the vehicle‐treated mice, but all these tissues were absent in metformin‐treated mice (Figure 6A). However, the bone mineral density and trabecular number of ectopic bone were the same in the treated and untreated groups (data not shown). In addition, we examined AMPK phosphorylation and Smad6 in the muscles around the Achilles tendon. Metformin treatment significantly increased AMPK phosphorylation and the level of Smad6 and Smurf1, concurrently with the reduction of ALK2 (Figure 6B&C). All these results demonstrate that metformin significantly reduces HO development following the Achilles tenotomy and burn.

## 4 DISCUSSION

In this study, we showed that metformin inhibited BMP signalling through up‐regulation of Smad6. The changes were associated with the inhibition of osteogenic differentiation. Second, we found that the effect of metformin was mimicked by the constitutively active mutant of AMPK and blocked by its dominant negative mutant or knockdown of Smad6. Finally, our study revealed that metformin prevented HO induced by traumatic injury in a mouse model, which reliably generated ectopic bone within soft tissues. The finding of increased phosphorylation of AMPK, up‐regulated Smad6 and decreased ALK2 in the injured tendons suggest that metformin reduces HO via AMPK regulation of Smad6, leading to inhibition of Smad1/5.

Trauma‐induced HO has been associated with inflammation in the local injured area, where BMPs are secreted and activate osteogenic programme including chondrogenesis and osteogenesis.^19^, ^33^ Thus, therapeutic strategy has been pursued to target BMP signalling pathway. Blockade of active BMP signalling pathway effectively mitigates HO progression in different animal models.^25^, ^26^, ^27^, ^33^ For example, inhibition of BMP signalling with A3Fc and LDN21 reduces trauma‐induced HO in mice.^25^ A3Fc is a receptor trap protein derived from ALK3 containing the BMP‐binding region and exhibits less adverse effect without myelosuppression and immunosuppression. Treatment with adenosine triphosphate (ATP) hydrolysis apyrase prevents HO through inhibition of the BMP signalling pathway.^26^ Here, we showed that metformin reduced HO formation by activation of AMPK and subsequent inhibition of the BMP signalling pathway. The present findings are in line with our previous study showing that metformin inhibits Smad1/5 signalling downstream of the active mutant of ALK2 (R206H) identified in FOP patents in association with reduced osteogenesis *in vitro*.^28^ As metformin is widely recognized as a safe and inexpensive drug, it will be attractive to repurpose its clinical application to the prevention and treatment of HO.

It has been controversial about the role of AMPK in osteogenesis and osteoclasterosis.^34^, ^35^, ^36^ The discrepant findings might be attributable to differences in cell context and settings. Osteogenesis is a process that requires a large amount of ATP and glucose supply for anabolism, which leads to mTOR activation and AMPK inhibition, the two opposite fuel‐sensing systems.^37^, ^38^ In keeping with this notion, previous and our present studies have shown that AMPK activity is suppressed during osteoblast differentiation and AMPK activation by metformin or expression of constitutive active mutant of AMPK blocks the differentiation. However, metformin still suppressed differentiation in the presence of dominant negative of AMPK. This suggests two possibilities. First, exogenously expressed dominant negative mutant of AMPK was not sufficient to completely compete with endogenous wild‐type AMPK. Second, metformin acts through both AMPK‐dependent and independent mechanisms. Interestingly, previous studies have reported that a well‐received downstream target of AMPK, mTOR, participates in regulation of osteogenic differentiation and trauma‐induced HO, whereas inhibition of mTOR with rapamycin suppresses HO in animal models.^39^, ^40^ In considering the inhibitory effect of metformin/AMPK on mTOR, it is understandable that the same outcomes could be produced by metformin and rapamycin. Moreover, regarding clinical use, metformin is far more tolerable than rapamycin.

Recently, we have shown that AMPK up‐regulates Smad6 and Smurf1, leading to proteasomal degradation of ALK2,^28^ whereas the present study that AMPK up‐regulates Smad6 without significant changes in Smurf1 and ALK2. The common denominator is the increase of Smad6 by metformin. The different results may be attributable to differences in cell context. It has been documented that Smad6 preferentially inhibits BMP signalling pathway through the following mechanisms: (a) preventing phosphorylation of Smad1/5/8 by type I receptor, (b) disrupting the interaction with Smad4 and Smad1/5/8, and (c) promoting degradation of the receptor I, Smad1/5, or Runx2 by recruiting Smurf1, an E3 ubiquitin ligase. Therefore, it is plausible that a different mechanism may specifically prevail in one cell type, regardless that same outcome is generated as to the inhibition of BMP signalling. In keeping with this notion, the results from our animal model showed the induction of both Smad6 and Smurf1 and down‐regulation of ALK2 by metformin, whereas only Smad6 was induced in MC3T3‐E1 cells. Collectively, our results suggest that Smad6 plays an essential role in mediating metformin‐induced inhibition of HO.

Development of HO in the Achilles tenotomy mouse model occurs through endochondral ossification, which involves in multiple stages including inflammation, cellular infiltration, mesenchymal condensation, chondrogenesis and osteogenesis, eventually form ectopic bones.^41^, ^42^ Our study showed that metformin prevented HO, possibly through inhibition of the BMP signalling pathway. However, we do not know if metformin acts at chondrogenesis or osteogenesis as it was administered immediately after injury. Since BMP/ALK2 signalling has been implicated in the whole HO process,^43^ it is possible that metformin could interfere with both chondrogenesis and osteogenesis.

The premise to use metformin to suppress HO is that it does not affect normal fracture healing process as the two often concur. Previous studies have reported that type 2 diabetes mellitus (T2DM) exhibits increased risk of bone fracture,^44^, ^45^, ^46^ whereas metformin does not have an effect^47^, ^48^, ^49^ or reduces the fracture in diabetic patients.^50^, ^51^ Furthermore, studies using animal model have shown that metformin does not have negative impact on fracture healing with metformin.^52^, ^53^ This indicates that metformin does not affect normal bone repair via intramembranous or endochondral ossification process. Therefore, it would be safe to use metformin in prophylaxis of HO in the case of fracture.

Matrix metalloproteinase‑9 (MMP9) plays a critical role in endochondral ossification and bone formation under both physiological and pathological conditions.^54^, ^55^ MMP‐9 activity is significantly up‐regulated in the early stage of HO induced by Achilles tenotomy in rat and BMP in mice.^56^, ^57^, ^58^ Inhibition of MMP‐9 activity by minocycline effectively prevented the HO formation in mice.^57^Several studies have showed that AMPK negatively regulates MMP‐9 expression and activity.^55^, ^59^, ^60^Our data revealed that metformin attenuated HO in mice and induced activation of AMPK. It is possible that administration of metformin suppressed MMP9 expression via activation of AMPK, leading to prevention of HO. It will be interesting to investigate whether MMP‐9 plays a role in mediating the effect of metformin/AMPK on HO.

In the end, we should point out that although we did not have direct evidence that metformin through inhibition of BMP/ALK2 attenuates HO in animal model, we observed increased expression of Smad6 and decreased abundance of ALK2 in association with AMPK activation. Therefore, we are confident that metformin through AMPK‐Smad6 regulatory axis suppresses HO. Thus, our study supports the rationale to retask metformin for prevention and treatment of HO. As it is cheap and safe, it would have a great potential in clinical use for this disease.

## CONFLICT OF INTEREST

The authors declare that they have no competing interests.

## AUTHOR CONTRIBUTIONS

**Hui Lin:** Conceptualization (equal); Formal analysis (equal); Investigation (equal); Methodology (equal); Validation (equal); Visualization (equal); Writing‐original draft (equal). **Fuli Shi:** Conceptualization (equal); Data curation (equal); Investigation (equal); Software (equal). **Shanshan Jiang:** Methodology (equal); Resources (equal); Software (equal). **Yuanyuan Wang:** Conceptualization (equal); Data curation (supporting); Investigation (supporting). **Junrong Zou:** Resources (supporting); Software (supporting). **Ying Ying:** Conceptualization (supporting); Formal analysis (supporting); Investigation (supporting). **Deqiang Huang:** Methodology (supporting); Resources (supporting); Validation (supporting). **Lingyu Luo:** Methodology (supporting); Resources (supporting); Software (supporting). **Xiaohua Yan:** Resources (supporting); Software (supporting). **Zhijun Luo:** Conceptualization (equal); Investigation (equal); Project administration (equal); Supervision (lead); Writing‐original draft (lead); Writing‐review & editing (lead).

## Supporting information

Fig S1

## Data Availability

The data that support the findings of this study are available from the corresponding author upon reasonable request.
