# Supplementary figures and images for: Metformin attenuates trauma‐induced heterotopic ossification via inhibition of Bone Morphogenetic Protein signalling
Source: J Cell Mol Med. 2020 Nov 10;24(24):14491–501. doi: 10.1111/jcmm.16076 (PMC7754007; doi:10.1111/jcmm.16076)

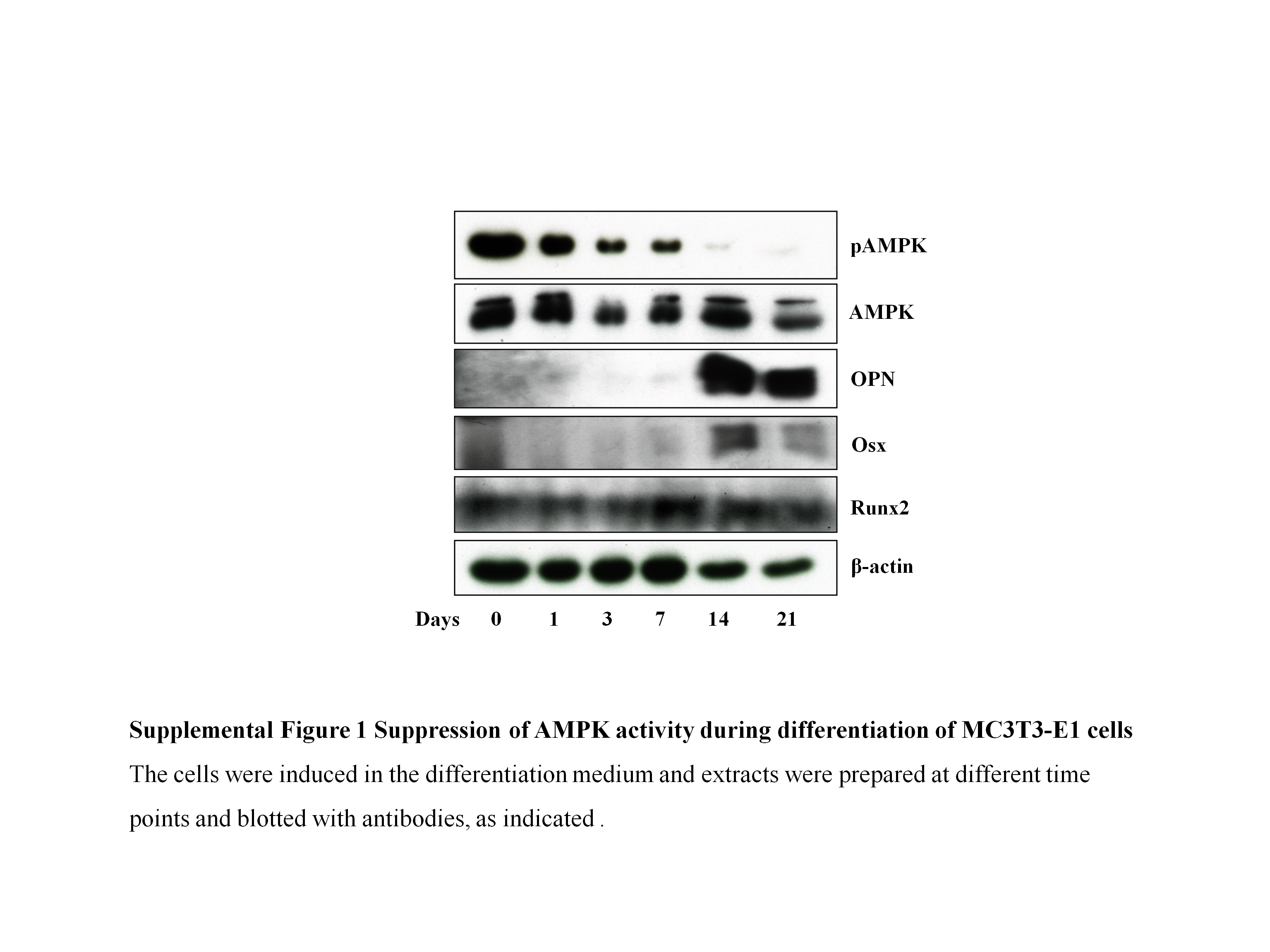

Supplement: Supplementary file 1 — Fig S1 [file JCMM-24-14491-s001.tif]
